# Mesenchymal Stem Cell-Derived Exosomes Attenuate Murine Cytomegalovirus-Infected Pneumonia via NF-κB/NLRP3 Signaling Pathway

**DOI:** 10.3390/v16040619

**Published:** 2024-04-16

**Authors:** Fei Chen, Zhida Chen, Hui-Ting Wu, Xin-Xiang Chen, Peiqi Zhan, Zheng-Yi Wei, Zizhang Ouyang, Xueyan Jiang, Ao Shen, Min-Hua Luo, Qifa Liu, Yue-Peng Zhou, Aiping Qin

**Affiliations:** 1 Guangzhou Municipal and Guangdong Provincial Key Laboratory of Molecular Target & Clinical Pharmacology, The NMPA and State Key Laboratory of Respiratory Disease, School of Pharmaceutical Sciences and the Sixth Affiliated Hospital, School of Basic Medical Science, Guangzhou Medical University, Guangzhou 511436, China; chenfei@gzhmu.edu.cn (F.C.); chenzhida1998@163.com (Z.C.); wuhuiting2000@163.com (H.-T.W.); xinxiang8375@163.com (X.-X.C.); 13532826402@163.com (P.Z.); 13724372709@163.com (Z.-Y.W.); xjiang@gzhmu.edu.cn (X.J.); shenao@gzhmu.edu.cn (A.S.); 2 Affiliated Cancer Hospital and Institute of Guangzhou Medical University, Guangzhou Municipal and Guangdong Provincial Key Laboratory of Protein Modification and Degradation, Center for Cancer Research and Translational Medicine, School of Basic Medical Sciences, Guangzhou Medical University, Guangzhou 511436, China; 3 Department of Pharmaceutical Sciences, The Sixth Affiliated Hospital of Guangzhou Medical University, Qingyuan People’s Hospital, Qingyuan 511518, China; oyzz8100@126.com; 4 State Key Laboratory of Virology, CAS Center for Excellence in Brain Science and Intelligence Technology (CEBSIT), Wuhan Institute of Virology, Chinese Academy of Sciences, Wuhan 430071, China; luomh@wh.iov.cn; 5 Department of Hematology, Nanfang Hospital, Southern Medical University, Guangzhou 510515, China; 6 State Key Laboratory of Magnetic Resonance and Atomic and Molecular Physics, National Center for Magnetic Resonance in Wuhan, Wuhan Institute of Physics and Mathematics, Wuhan 430071, China

**Keywords:** mesenchymal stem cell, exosome, murine cytomegalovirus, pneumonia, NLRP3 inflammasome

## Abstract

Reactivation and infection with cytomegalovirus (CMV) are frequently observed in recipients of solid organ transplants, bone marrow transplants, and individuals with HIV infection. This presents an increasing risk of allograft rejection, opportunistic infection, graft failure, and patient mortality. Among immunocompromised hosts, interstitial pneumonia is the most critical clinical manifestation of CMV infection. Recent studies have demonstrated the potential therapeutic benefits of exosomes derived from mesenchymal stem cells (MSC-exos) in preclinical models of acute lung injury, including pneumonia, ARDS, and sepsis. However, the role of MSC-exos in the pathogenesis of infectious viral diseases, such as CMV pneumonia, remains unclear. In a mouse model of murine CMV-induced pneumonia, we observed that intravenous administration of mouse MSC (mMSC)-exos reduced lung damage, decreased the hyperinflammatory response, and shifted macrophage polarization from the M1 to the M2 phenotype. Treatment with mMSC-exos also significantly reduced the infiltration of inflammatory cells and pulmonary fibrosis. Furthermore, in vitro studies revealed that mMSC-exos reversed the hyperinflammatory phenotype of bone marrow-derived macrophages infected with murine CMV. Mechanistically, mMSC-exos treatment decreased activation of the NF-κB/NLRP3 signaling pathway both in vivo and in vitro. In summary, our findings indicate that mMSC-exo treatment is effective in severe CMV pneumonia by reducing lung inflammation and fibrosis through the NF-κB/NLRP3 signaling pathway, thus providing promising therapeutic potential for clinical CMV infection.

## 1. Introduction

Cytomegalovirus (CMV) infection is often asymptomatic in individuals with a normal immune system. However, individuals with compromised immune systems, such as transplant recipients and AIDS patients, may experience symptoms when CMV reactivates or during primary infection. These symptoms can include fever, bone marrow suppression, and tissue-invasive disease like pneumonitis [1]. CMV pneumonia is a major cause of death following allogeneic bone marrow transplantation [2]. Currently, antiviral drugs like valganciclovir or ganciclovir are the preferred first-line treatments for CMV infections [3]. However, the effectiveness of these treatments is not ideal, and further exploration of alternative approaches is necessary. To investigate immune responses and therapeutic strategies for CMV infection, murine CMV (MCMV) is commonly used as a model for human CMV (hCMV) diseases due to their similar pathogenesis in the lungs and other organs [4,5]. 

Interstitial lung inflammation is a characteristic feature of several CMV infections in patients [6]. CMV interacts with its host immune system and contributes to immune activation and hyperinflammation. CMV infection activates the NF-κB and NLRP3 signaling pathways, promoting the production of various pro-inflammatory mediators, including interleukin-1β (IL-1β), IL-6, tumor necrosis factor alpha (TNFα), and interferons [7,8]. These inflammatory mediators play crucial roles in the inflammatory process underlying lung tissue injury [9]. The mechanisms by which CMV regulates inflammation are not fully understood. It is known that the activation of immune cells provides critical cell-derived transcription factors, such as NF-κB- and cAMP-derived transcription factors, which are important for CMV replication [8,10]. Therefore, modulating the inflammatory signaling pathway may be crucial for improving CMV pneumonitis.

An emerging therapeutic approach for the treatment of pneumonia involves the use of exosomes derived from mesenchymal stem cells (MSCs). These exosomes have gained attention due to their potential to effectively reverse the symptoms of pneumonia [11,12,13], lung injury [14,15], acute respiratory distress syndrome (ARDS) [16], chronic obstructive pulmonary disease (COPD) [17], bronchial asthma [18], and idiopathic pulmonary fibrosis [19]. Exosomes are small extracellular vesicles (EVs) that contain various bioactive molecules, including proteins, lipids, and nucleic acids. They play a crucial role in intercellular communication [20,21]. Exosomes derived from MSCs have demonstrated anti-inflammatory, immunomodulatory, and regenerative properties, making them a promising tool for treating pulmonary diseases [11,12,22]. However, the role of MSC-secreted exosomes in viral illnesses, such as CMV pneumonitis, remains uncertain. Further investigation is needed to fully understand the potential therapeutic effects and molecular mechanisms of MSC-derived exosomes in this context.

In this study, the authors hypothesize that mMSC-secreted exosomes may effectively treat MCMV pneumonia by attenuating inflammatory response. This mechanism may involve the modulation of macrophage phenotype from M1 to M2 and the suppression of the NF-κB/NLRP3 signaling pathway. 

## 2. Materials and Methods

### 2.1. Mice 

C57Bl/6J germ-free mice (4–6 weeks old) and female BALB/c nude mice (4–6 weeks old) were obtained from Beijing Huafukang Biotechnology Co., Ltd. (Beijing, China) and housed in a pathogen-free mouse room at the experimental animal center of Guangzhou Medical University. The Animal Ethics Committee of Guangzhou Medical University approved all animal-related experimental procedures (Ethics number: 2018-132).

### 2.2. Cell Culture

The murine fibroblast cell line (NIH/3T3) was obtained from the American Type Culture Collection (Manassas, VA, USA). The 3T3 cells were cultured in Dulbecco’s modified Eagle’s medium (DMEM; Gibco, Invitrogen, Carlsbad, CA, USA) supplemented with 10% fetal bovine serum (FBS; Gibco), 2 mM glutamine, and 100 IU/mL penicillin/streptomycin.

Mouse MSCs were isolated, cultured, and characterized as follows. Under aseptic conditions, bone marrow was extracted from C57BL/6 mice aged 4–6 weeks. Femurs and tibiae were collected and placed on ice in 4–5 mL of low-glucose DMEM (L-DMEM; Gibco). The bone marrow cavities were flushed with the medium, and a single-cell suspension was obtained by filtering the collected liquid through a 70 μm cell strainer. Ammonium chloride lysis was used to remove red blood cells, and the remaining cells were resuspended and cultured in L-DMEM complete medium with 10% FBS at 37 °C and 5% CO_2_. After three days, nonadherent cells were discarded, and the adherent cells were further cultured for 10–14 days, forming homogeneous fibroblast-like colonies. The cells at 80% confluence were detached using 0.25% trypsin–EDTA and labeled as passage no. 1. Subsequent passages were performed at a 1:3 ratio. The expression of surface markers in the MSCs was analyzed by flow cytometry analysis (Beckman Coulter, Brea, CA, USA) using CD29 (Integrin beta 1) antibody (#17-0291-80, Clone: eBioHMb1-1 (HMb1-1); eBioscience, San Diego, CA, USA), CD44 monoclonal antibody (IM7) (#12-0441-81, Clone: IM7; eBioscience), Ly-6A/E (Sca-1) monoclonal antibody (D7) (#45-5981-80, Clone: D7; eBioscience), CD105 (Endoglin) monoclonal antibody (MJ7/18) (#12-1051-81, Clone: MJ7/18; eBioscience), CD34 monoclonal antibody (RAM34) (#11-0341-82, Clone: RAM34; eBioscience), and APC/Cyanine7 anti-mouse CD45 antibody (#103116, clone: 30-F11; BioLegend, San Diego, CA, USA). The differentiation potential of MSCs into osteoblasts and adipocytes was evaluated using Oil Red O and Alizarin Red S staining, respectively. The MSCs from passages 3 and 8 were chosen for analysis.

#### 2.2.1. Identification of Exosomes from 3T3 and mMSCs 

Exosomes were isolated from both 3T3 and mMSCs using a standardized protocol. The cells were cultured in exosome-depleted media and then prepared by subjecting complete growth media containing 10% FBS to ultracentrifugation at 100,000× *g* for 16 h at 4 °C. The resulting supernatant was collected and filtered through a 0.22-μm filter to remove any remaining debris.

The 3T3 and mMSCs were seeded at a density of 5 × 10^4^ cells/cm2 and cultured in exosome-depleted media for 48 h. After the incubation period, the conditioned media were collected and subjected to a series of centrifugation steps to remove cells, cell debris, and large vesicles. These steps included an initial centrifugation at 300× *g* for 10 min, followed by 2000× *g* for 20 min, and then 10,000× *g* for 30 min at 4 °C. The resulting supernatants were then ultracentrifuged at 100,000× *g* for 70 min at 4 °C to pellet the exosomes. The exosome pellet was subsequently washed in PBS and underwent another round of ultracentrifugation at 100,000× *g* for 70 min at 4 °C.

The resulting exosome pellet was resuspended in an appropriate volume of PBS and stored at −80 °C for further analysis. The protein concentration of the isolated exosomes was quantified using a BCA protein assay (Pierce, Thermo Fisher Scientific, Rockford, IL, USA) according to the manufacturer’s instructions. The morphology, size distribution, and presence of exosomal markers (such as CD63, Alix, and TSG 101) were assessed using transmission electron microscopy and Western blotting to confirm the successful isolation of exosomes from 3T3 and mMSC cells.

#### 2.2.2. Viral Plaque Assay and Viral Infections 

Dr. Min-hua Luo from the Wuhan Institute of Virology, China generously provided the MCMV-GFP (Smith strain). The MCMV was propagated and purified utilizing 3T3 cells. NIH/3T3 cells were cultured in 15 cm dishes containing complete DMEM and transfected with MCMV at a multiplicity of infection (MOI) of 0.1 for 3–4 days. The culture supernatants were filtered through a 0.45 µm filter, and the virus particles were pelleted by ultracentrifugation over a 30% sucrose cushion at 25,000 rpm for 3 h at 4 °C, utilizing the Beckman SW32Ti rotor. Virus aliquots were stored at −80 °C, and the viral titers were determined by performing a plaque assay in NIH/3T3 cells. Infectious viruses (expressed as plaque-forming units (PFUs)) were quantified through serial log10 dilutions of virus stocks. The 3T3 cells were cultured overnight in 24-well plates at a density of 1 × 10^5^ cells per well and infected with various virus dilutions for 3–6 h. The cell medium was replaced with an overlay medium containing agarose, and cells were then incubated at 37 °C for 4–5 days. PFU counts per well were determined using a Leica (DMi8) microscope (Leica, Wetzlar, Germany) once the fluorophore became visible.

For in vivo infections, the frozen MCMV sample was thawed on ice. Mice were infected intranasally with either MCMV (2 × 10^5^ PFU) or PBS vehicle control and housed in microisolator cages with ad libitum access to food and water. Mouse body weight and survival were monitored and recorded every two days post infection.

For in vitro infections, BMDMs were treated with MCMV at an MOI of 0.05 for 48 h. Supernatants were collected to determine viral titers through PFU assay and to characterize cytokine expression using ELISA. Cells were collected for Western blot and flow cytometry analyses.

#### 2.2.3. Bronchoalveolar Lavage (BAL)

Three days after MCMV infection, each mouse was euthanized, and its trachea was exposed. A 20-gauge angiocatheter was inserted into the trachea, and the lungs were flushed three times with 0.8 mL of sterile PBS. The bronchoalveolar lavage (BAL) fluid was collected, pooled, and then centrifuged at 500× *g* for 5 min at 4 °C to separate the cellular fraction. The resulting BAL fluid was stored at −80 °C for PFU and ELISA assays, while the cell pellet was resuspended in cold PBS for flow cytometry analysis (Beckman Coulter, USA).

#### 2.2.4. FACS Analysis 

Surface markers of bone marrow-derived mesenchymal stem cells (BMSCs) and cell types from bronchoalveolar lavage (BAL) fluid were assessed using flow cytometry using Ly-6G monoclonal antibody (1A8-Ly6g) (#25-9668-82, Clone: 1A8-Ly6g; eBioscience), F4/80 monoclonal antibody (BM8) (#12-4801-82, Clone: BM8; eBioscience), and CD11b monoclonal antibody (M1/70) (#48-0112-82, Clone: M1/70; eBioscience). BAL cells were obtained by centrifuging pooled BAL fluid, and the resulting cell pellet was stained with fluorochrome-labeled antibodies to determine leukocyte differential, including neutrophils (CD11b+Ly6G+), macrophages (CD11b+F4/80+), and monocytes (CD11b+Ly6C+). 

Total cell numbers were determined using a cell counter (Jimbio, Changzhou, Jiangsu, China) by analyzing the BAL cell pellet prior to flow cytometry, following the manufacturer’s instructions. The surface markers of M1 or M2 macrophages from BAL and bone marrow-derived macrophages (BMDMs) were also measured using CD80 (B7-1) monoclonal antibody (16-10A1) (11-0801-81, Clone:16-10A1; eBioscience) and CD206 (MMR) monoclonal antibody (MR6F3) (17-2061-80, Clone:MR6F3; eBioscience). Isotype and secondary-only controls were used to control potential nonspecific staining due to Fc–receptor interactions. Data analysis was conducted using FACSCanto II (Beckman Biosciences, Brea, CA, USA) and FlowJo 10 software (Tree Star, Ashland, OR, USA). Further analyses were performed using CytoFLEX (Beckman Biosciences) and FlowJo 7.6 software (Tree Star, Ashland, OR, USA).

#### 2.2.5. Histological Analysis and Immunofluorescence Staining 

The lungs, which were not trimmed or inflated, were extracted from the animals and fixed in a 4% buffered paraformaldehyde solution. Subsequently, the lungs were embedded in paraffin. Sections with a thickness of 4 µm were then cut and processed for Hematoxylin/Eosin (H&E) or Masson staining, as well as immunofluorescence staining. Both staining procedures were conducted by Servicebio in Wuhan, China. The H&E-stained slides were examined for lung pathology induced by the virus using a Leica (DMi8) microscope. The percentage of airspace and acinar tissue was determined in each slide from five randomly selected animals in each mouse group using ImageJ software Version 1.54. For each mouse, 30 high-power fields were randomly selected and assessed, with the investigators blinded to the animal group allocation. Lung fibrosis severity was assessed on a scale ranging from 0 to 8 by examining randomly selected sections [23]. The criteria for grading lung fibrosis were as follows: grade 0 represented a normal lung, grade 1 indicated minimal fibrous thickening of alveolar or bronchiolar walls, grade 3 denoted moderate thickening of walls without evident damage to lung architecture, grade 5 indicated increased fibrosis with definitive damage to lung structure and the presence of fibrous bands or small fibrous masses, grade 7 represented severe distortion of structure and the presence of large fibrous areas, and grade 8 indicated complete fibrous obliteration of fields.

For immunofluorescence staining, the sections were mounted on glass slides (Bio-Optica, Milan, Italy). They were then blocked with 0.05% PBS-Tween and 0.5% FBS for 30 min. Next, the sections were incubated at 4 °C overnight with a combination of primary antibodies against α-SMA (1:500, #19245; CST, Danvers, MA, USA), collagen I (1:500, #AF7001; Affinity, Cincinnati, OH, USA), NLRP3 (1:500, DF7438; Affinity), and ASC (1:500, #sc-514414; SCBT, Dallas, TX, USA). After incubation, the sections were washed three times with PBS and incubated at room temperature for 1 h with a combination of Alexa Fluor 488-conjugated Goat Anti-Rabbit IgG (H + L) (1:500, #GB21303; Servicebio, Wuhan, Hubei, China) or Alexa Fluor 647-conjugated Goat Anti-Mouse IgG (H + L) (1:500, #GB22301; Servicebio). The sections were then washed again and counterstained with DAPI (4′,6-diamidino-2-phenylindole). Finally, they were mounted in Prolong Gold antifade reagent (Life Technologies, Carlsbad, CA, USA). Fluorescence was visualized using a Zeiss LCM880 confocal microscope (Oberkochen, Germany) and analyzed using Zen or ImageJ software (NIH, Bethesda, MD, USA). 

#### 2.2.6. Enzyme-Linked Immunosorbent Assay (ELISA) 

The excised lungs were weighed, washed, and homogenized in PBS. The expression of cytokines and chemokines, namely TNF-α, IL-10, IL-1β, monocyte chemoattractant protein-1 (MCP-1) (Neobioscience, Beijing, China), and C-X-C chemokine receptor type 1 (CXCR1) (R&D Systems), was evaluated in the supernatants obtained from lung homogenates and BAL fluid. ELISA was employed to quantify the levels of these proteins, following the guidelines provided by the manufacturer.

#### 2.2.7. Western Blot Analysis 

Immunoblotting was conducted following standard procedures [24]. Lung tissues or BMDM samples were briefly lysed using a radio-immunoprecipitation assay (RIPA) buffer containing a protease inhibitor mixture. The supernatants were collected and quantified using a BCA protein quantification kit (ThermoFisher Scientific, Rockford, IL, USA). The lysates were then boiled for 10 min, and equal amounts of proteins were separated by SDS-PAGE and transferred to a polyvinylidene fluoride (PVDF) membrane (Immobilon, Burlington, MA, USA). The membrane was blocked and incubated with the primary antibody overnight at 4 °C. Subsequently, a secondary antibody conjugated with horseradish peroxidase was applied, and the membrane was exposed to room temperature for 1 h. β-tubulin was used as a loading control. Protein levels were assessed using the following primary antibodies: NF-κB (1:1000, #8242; CST), pNF-κB (1:1000, #3033; CST), NLRP3 (1:1000, #DF7438; Affinity), ASC (1:500, #sc-514414; SCBT), Caspase1 (1:1000, #AF5418; Affinity), CollagenI (1:1000, #AF7001; Affinity), α-SMA (1:1000, #19245; CST), TSG101 (1:1000, #sc-528189; SCBT), Alix (1:1000, #67715-1-IG; Proteintech, Rosemont, IL, USA), CD63 (1:1000, #sc-5275; SCBT), β-tubulin (1:2000, #T0023; Affinity), IL-1β (1:1000, AF5103), ARG1 (1:1000, #DF6657; Affinity), and INOS (1:1000, #AF0199; Affinity). Antigen–antibody complexes were detected using enhanced chemiluminescence (GE Amersham Imager 600, Chicago, IL, USA). Band intensities were quantified using ImageJ software(Version 1.54) and standardized based on β-tubulin levels. 

#### 2.2.8. Statistical Analysis

All statistical analyses were conducted using GraphPad Prism version 7.0. Significance between multiple groups was determined through one-way analysis of variance (ANOVA), followed by post hoc group-wise comparisons using Tukey’s test. Survival data were represented as Kaplan–Meier curves and assessed for statistical significance using the Mantel–Cox test. Quantitative data were presented as mean ± SEM, and significant differences were defined as * *p* < 0.05, ** *p* < 0.01, *** *p* < 0.001, and **** *p* < 0.0001.

## 3. Results

### 3.1. Characterization of mMSC-exos 

**Figure 1:**
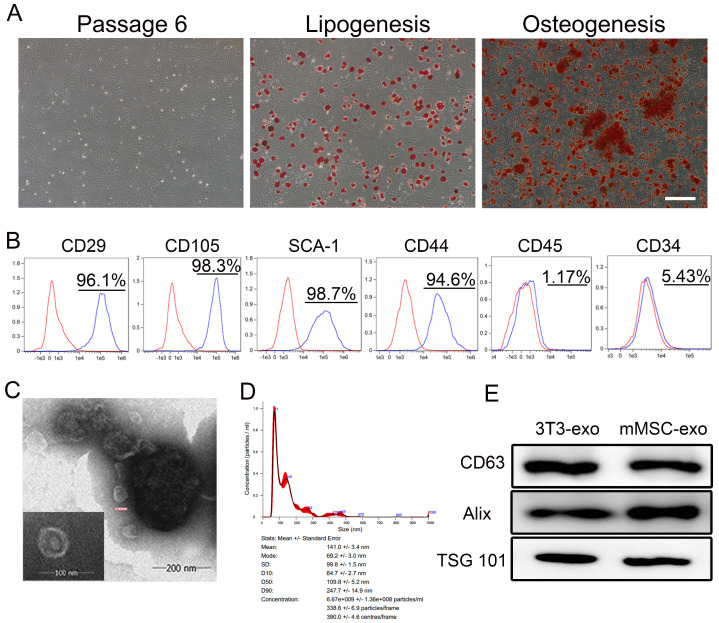
Characterization of mMSC-exos. (**A**) The morphology of mMSCs was monitored under a microscope at passage 6, and these mMSCs can be successfully induced into adipocytes or osteoblasts. Oil Red O staining shows lipid droplets and Alizarin Red S staining shows calcium content in cells. The scale bar indicates 200 μm. (**B**) Flow cytometric analysis of the mMSC-determining surface markers (CD29, CD105, SCA−1, CD44) and hematopoietic cell markers (CD45 and CD34) on mMSC. Red: isotype control; Blue: specific antibody. (**C**) Representative TEM images presenting the morphology of mMSC-exos. (**D**) Size distribution of mMSC-exos. (**E**) Western blotting of protein expression in 3T3-exos and mMSC-exos.

The primary step in our study involved assessing the differentiation capacity and phenotypic markers of the mMSCs. Successful induction of adipocyte and osteoblast differentiation confirmed that the mMSCs exhibited MSC characteristics (Figure 1A). Additionally, flow cytometry analysis demonstrated a strong expression of MSC markers (CD29, CD105, CD44, and SCA-1) in the isolated mMSCs, while hematopoietic cell markers (CD34 and CD45) were not detected (Figure 1B).

The size and morphology of the mMSC-exos were confirmed through TEM analysis (Figure 1C). Size distribution histograms were generated from 24 h serum-free conditioned media (Figure 1D). Immunoblot analyses confirmed the expression of exosome core markers CD63, Alix, and TSG 101 (Figure 1E). These findings demonstrate that all mMSC populations produce and release EVs with typical exosome characteristics.

### 3.2. mMSC-exos Improved Survival and Reduced Lung Injury in MCMV-Infected Mice

**Figure 2:**
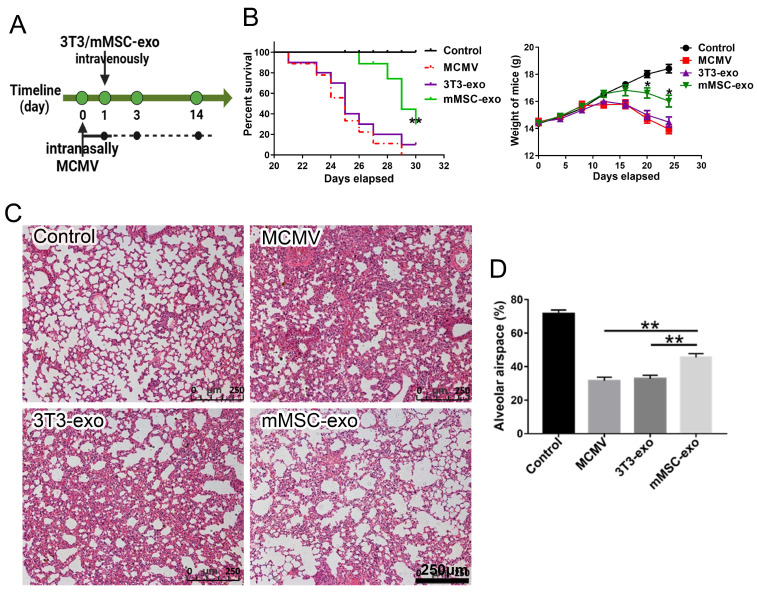
mMSC-exos improved survival and reduced lung injury in MCMV-infected mice. Groups of female BALB/c nude mice were infected with MCMV (2 × 10^5^ PFU) and treated with mMSC-exos (30 μg per mouse). (**A**) The timeline of the treatment. (**B**) Survival curves and body weight changes of MCMV-infected mice treated with mMSC-exos, up to 30 days after intranasal instillation of MCMV. *n* = 10. (**C**) Representative histological images of lung tissues harvested from mice at 14 days post infection. Scale bars = 250 μm. (**D**) The percentages of alveolar tissue and alveolar airspace were calculated. *n* = 6. Multiple group comparisons: one-way ANOVA with Tukey’s post hoc test. Survival data: Kaplan–Meier curves with Mantel–Cox test. Data are reported as mean ± SEM. * *p* < 0.05, ** *p* < 0.01, compared to MCMV group.

To assess the therapeutic potential of exosomes derived from mMSCs in the context of MCMV pneumonia, we induced MCMV pneumonia in immunocompromised mice. As illustrated in Figure 2A, all mice succumbed to MCMV infection within 30 days. However, the administration of mMSC exosomes significantly ameliorated the clinical symptoms of pneumonia in mice, resulting in prolonged survival and reduced weight loss (Figure 2B). Histological analysis of lung tissue confirmed the presence of leukocyte infiltration, increased alveolar tissue, and reduced alveolar airspace in the MCMV-induced pneumonia mouse model (Figure 2C,D). In contrast, treatment with mMSC exosomes mitigated the pulmonary damage caused by MCMV compared to the control group (Figure 2C,D). These findings indicate that mMSC exosomes have the potential to attenuate pulmonary injury in mice with MCMV pneumonia.

### 3.3. mMSC-exos Suppressed the Inflammatory Response in MCMV-Infected Mice

**Figure 3:**
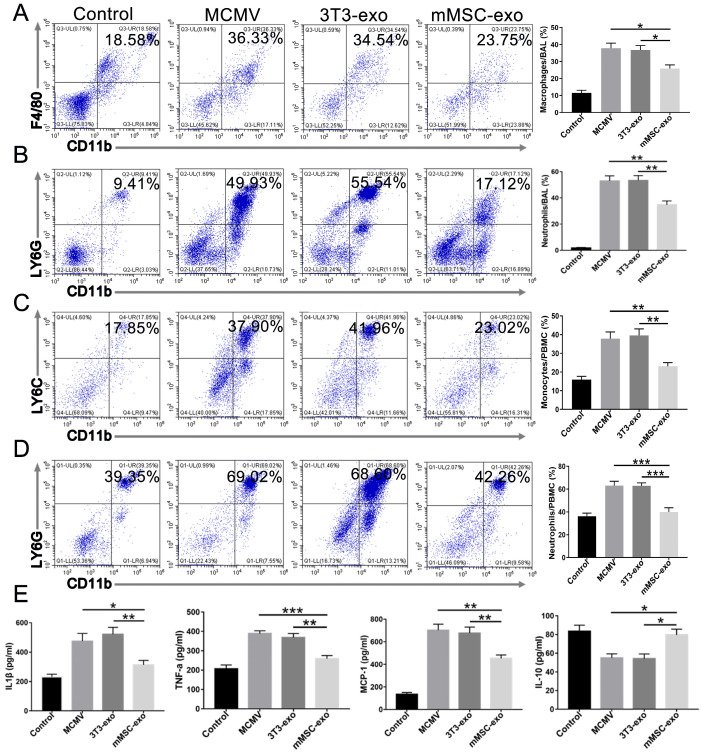
mMSC-exos suppressed the inflammatory response in MCMV-infected mice. Groups of female BALB/c nude mice were infected with MCMV (2 × 10^5^ PFU) and treated with mMSC-exos (30 μg per mouse). Representative flow cytometry plots and quantitative analysis of LY6G-CD11b+F4/80+ macrophages (**A**) and LY6G+CD11b+ neutrophils (**B**) in the collected BAL fluid of MCMV-infected mice treated with mMSC-exos at 3 days post-infection. Representative flow cytometry plots and quantitative analysis for LY6G-CD11b+Ly6C+ monocytes (**C**) and LY6G+CD11b+ neutrophils (**D**) in peripheral blood of MCMV-infected mice treated with mMSC-exos. (**E**) The levels of IL-1β, TNFα, MCP-1 and IL-10 in BAL fluid were detected by ELISA. Multiple group comparisons: one-way ANOVA with Tukey’s post hoc test. Data are reported as mean ± SEM, *n* = 6. * *p* < 0.05, ** *p* < 0.01, *** *p* < 0.001 compared to MCMV group.

CMV infection induces a systemic inflammatory response characterized by the recruitment of neutrophils and activation of macrophages [6,25,26]. We observed a significant expansion of CD11b+F4/80+ macrophages (gated on Ly6G- cells) and CD11b+Ly6G+ neutrophils in the lung BAL fluid following MCMV infection. However, this expansion was markedly reduced in the mMSC-exo group (Figure 3A,B). MCMV infection also resulted in a significant increase in the number of CD11b+Ly6C+ (gated on Ly6G- cells) monocytes and CD11b+Ly6G+ neutrophils in peripheral blood. In comparison to the model group, treatment with mMSC-exos clearly reduced the number of monocytes and neutrophils (Figure 3C,D). Figure 3E demonstrates that intratracheal instillation of MCMV led to a notable alteration in cytokine release in the BAL fluid. However, three days after infection, intravenous administration of mMSC-exos significantly reduced the levels of pro-inflammatory cytokines and chemokines, including TNF-α, IL-1β, and MCP-1, in the BAL fluid compared to treatment with MCMV and the 3T3-exo group. Additionally, mMSC-exos increased the secretion of the anti-inflammatory cytokine IL-10 in the BAL fluid (Figure 3E). These findings suggest that the administration of mMSC-exos may effectively alleviate lung inflammation caused by MCMV infection.

### 3.4. mMSC-exos Modulate Macrophage Polarization

According to the previous research, macrophages and monocytes are known to play a critical role in the production of inflammatory mediators during bacterial or viral infections, and they are also a primary target of MCMV infection [27]. Previous studies have shown that LPS-preconditioned MSC-exos can alleviate inflammation and regulate cytokine levels by modulating macrophage polarization in various inflammatory models [28]. Therefore, we aimed to investigate the potential of mMSC-exos in regulating macrophage polarization both in vivo and in vitro. 

**Figure 4:**
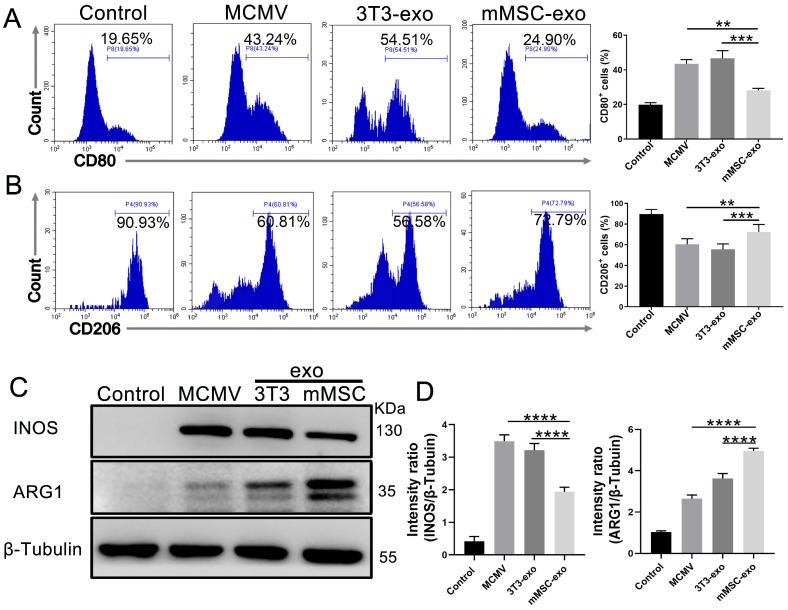
mMSC-exos modulate macrophage polarization in MCMV-infected mice. Groups of female BALB/c nude mice were infected with MCMV (2 × 10^5^ PFU) and treated with mMSC-exos (30 μg per mouse) for three days. Representative flow cytometry plots and quantitative analysis of the expressions of M1 macrophage marker CD80 (**A**) and M2 macrophage marker CD206 (**B**) in the collected BAL fluid of the mice. Gated on Ly6G-CD11b+F4/80+. (**C**) Western blotting of M1 macrophage marker INOS and M2 macrophage marker ARG1 in lung homogenates of MCMV-infected mice treated with mMSC-exos. (**D**) Proteins levels were calculated based on the signal intensities of the protein bands. Multiple group comparisons: one-way ANOVA with Tukey’s post hoc test. Data are reported as mean ± SEM, *n* = 6. ** *p* < 0.01, *** *p* < 0.001, **** *p* < 0.0001 compared to MCMV group.

In our in vivo study, we utilized flow cytometry to evaluate the expression of pulmonary M1/M2 markers (CD80 and CD206) after MCMV infection. The results demonstrated an increase in CD206 expression and a decrease in CD80 expression in BAL fluid from the mMSC-exo group (Figure 4A,B). Subsequently, we analyzed the expression of INOS and ARG1, which are well-known markers of M1 and M2 macrophage phenotypes, respectively, in lung homogenates on day 3 post-infection using Western blotting. The findings demonstrated an increase in M2 phenotypes and a decrease in M1 cells, as indicated by increased ARG1 expression and decreased INOS expression (Figure 4C,D). 

**Figure 5:**
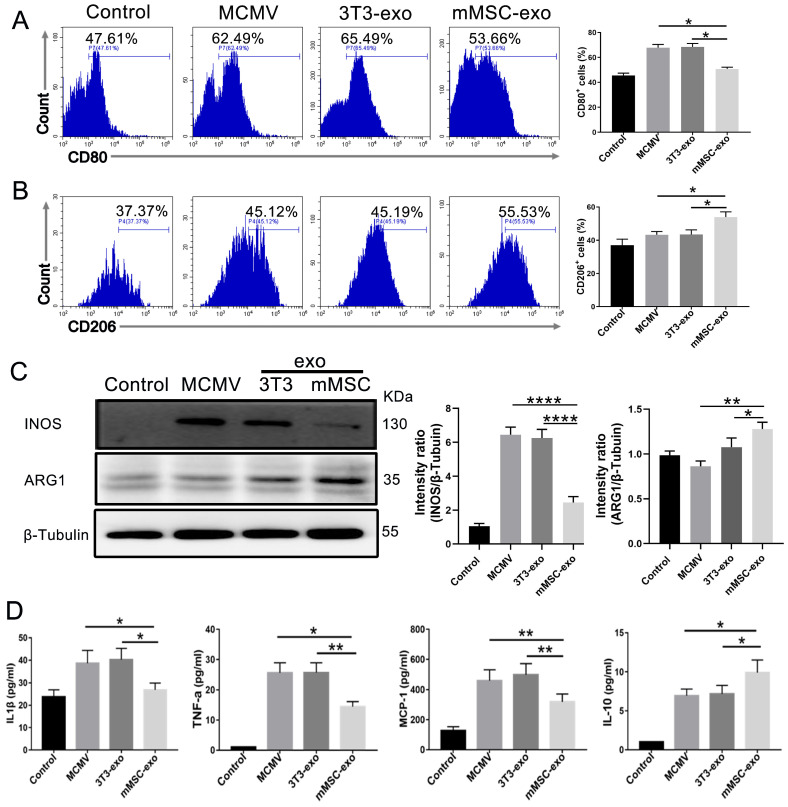
mMSC-exos modulate macrophage polarization in MCMV-infected BMDMs. Representative flow cytometry plots and quantitative analysis of the expressions of CD80 (**A**) and CD206 (**B**) in the MCMV-infected BMDMs. (**C**) Western blotting and quantitative analysis of INOS and ARG1 in MCMV-infected BMDMs. (**D**) ELISA was used to detect the levels of IL-1β, TNFα, MCP-1, and IL-10 in the cell supernatant. Multiple group comparisons: one-way ANOVA with Tukey’s post hoc test. Data are reported as mean ± SEM, *n* = 6. * *p* < 0.05, ** *p* < 0.01, **** *p* < 0.0001 compared to MCMV group.

Consistent with the in vivo findings, the administration of mMSC-exos in BMDM-infected MCMV resulted in a significant increase in CD206 and ARG1 expression and a significant decrease in CD80 and INOS expression (Figure 5A–C). Furthermore, the secretion of pro-inflammatory cytokines in the cell supernatant, such as TNF-α, IL-1β, and MCP-1, was reduced, while the anti-inflammatory cytokine IL-10 was elevated (Figure 5D).

These findings suggest that treatment with mMSC-exos can effectively alleviate lung inflammation by regulating macrophage polarization. 

### 3.5. mMSC-exos Reduce Pulmonary Fibrosis in MCMV-Infected Mice

**Figure 6:**
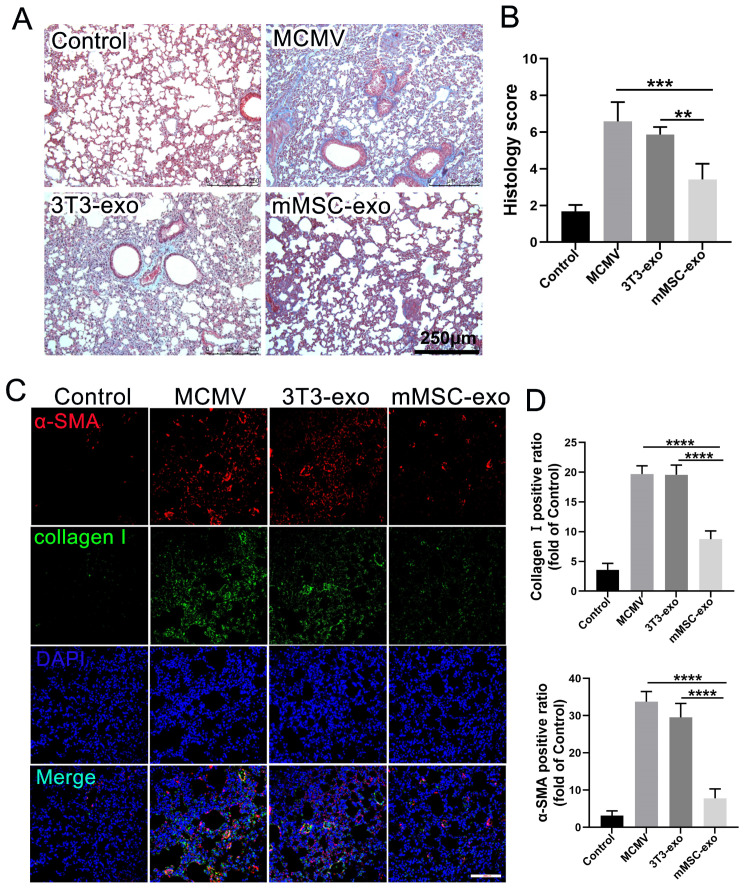
mMSC-exos reduce pulmonary fibrosis in MCMV-infected mice. (**A**) Masson’s trichrome staining was carried out to determine collagen levels in the lung tissue. The blue staining represents deposition of collagen. The severity of lung fibrosis was scored on a scale from 0 (normal lung) to 8 (total fibrotic obliteration of tissue in the examined fields). Scale bar: 250 μm. (**B**) Quantitative analysis of Masson staining by imageJ software. (**C**) The protein levels of α-SMA (red) and Collagen I (green) in lung tissues were measured by immunofluorescence staining. (**D**) Quantitative analysis of α-SMA and Collagen I positive areas in the lung. The integrated optical density values obtained from imageJ software were used to assess the expression of α-SMA and Collagen I in the stained sections. DAPI was used to nuclear staining (blue). Scale bars: 200 μm. Multiple group comparisons: one-way ANOVA with Tukey’s post hoc test. Data are expressed as mean ± SEM, *n* = 6. ** *p* < 0.01, *** *p* < 0.001, **** *p* < 0.0001 compared to MCMV group.

Previous research has indicated a correlation between active and long-term CMV infection and increased pulmonary fibrogenesis [29]. Therefore, we conducted an assessment of pulmonary fibrosis in mice infected with MCMV and explored the potential therapeutic effects of mMSC-exos. The administration of mMSC-exos resulted in a significant reduction in the severity of pulmonary fibrosis, as demonstrated by morphological changes observed through Masson’s trichrome staining on day 14 post-infection (Figure 6A,B). Additionally, the expression of α-SMA and Collagen I, which are prominent markers of pulmonary fibrosis, was notably decreased in the mMSC-exo mice compared to the MCMV-infected mice (Figure 6C,D). These findings suggest that mMSC-exos may have a beneficial therapeutic effect on pulmonary fibrosis in mice infected with MCMV.

### 3.6. mMSC-exos Inhibit NF-κB and NLRP3 Signaling Pathways

**Figure 7:**
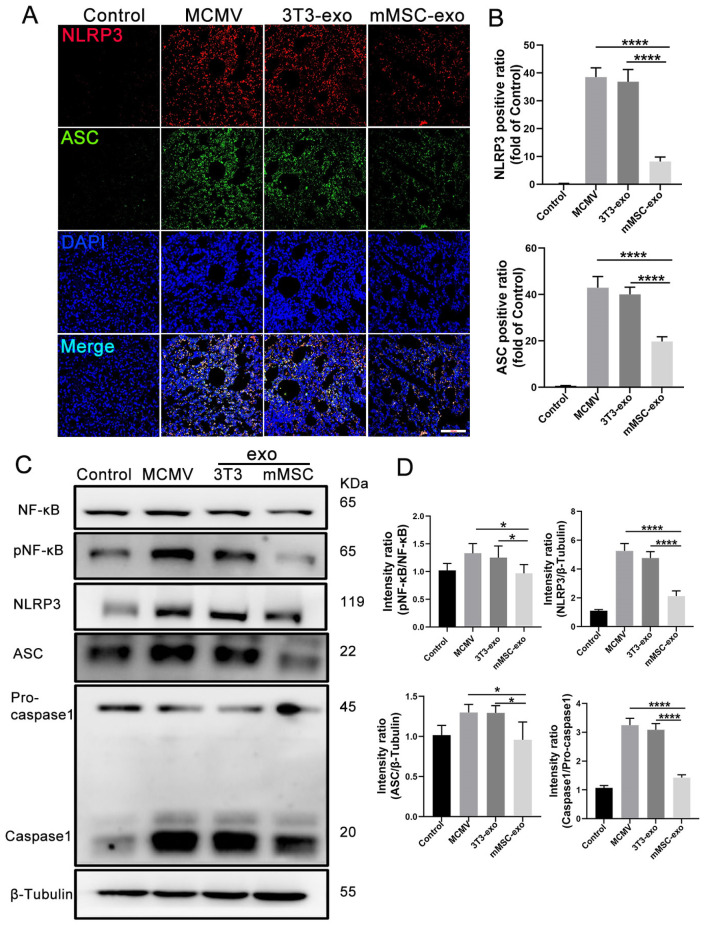
mMSC-exos inhibit NF-κB and NLRP3 signaling pathways in MCMV-induced pneumonia. (**A**) Representative images for NLRP3/ASC/DAPI immunofluorescence staining in the lung tissues were shown (NLRP3 = red, ASC = green, nuclei = blue); Scale bars: 200 μm. (**B**) Quantitative analysis of NLRP3 and ASC positive areas in the lung. The integrated optical density values obtained from image J software were used to assess the expression of NLRP3 and ASC in the stained sections. (**C**) Immunoblot analysis of NF-κB, pNF-κB, NLRP3, ASC, pro-caspase-1, and caspase-1. (**D**) Proteins levels were calculated based on the signal intensities of the protein bands. Multiple group comparisons: one-way ANOVA with Tukey’s post hoc test. Data are reported as mean ± SEM, *n* = 6. * *p* < 0.05, **** *p* < 0.0001 compared to MCMV group.

Previous studies have demonstrated the crucial role of the NF-κB and NLRP3 signaling pathways in the inflammatory response induced by CMV [7,8]. In order to investigate the impact of mMSC-exos on the activation of NLRP3, we evaluated the expression of relevant components within the NLRP3 signaling pathway. Immunofluorescence staining was conducted on day 3 post-infection to examine the expression of NLRP3 and ASC in lung tissue (Figure 7A,B). The assessment revealed a significant reduction in the protein expression of NLRP3 and ASC, indicating that mMSC-exos attenuated the activation of the NLRP3 inflammasome. This observation was further confirmed by Western blot analysis of lung homogenates, which showed decreased levels of NF-κB, pNF-κB, NLRP3, ASC, and caspase-1 in the mMSC-exo group (Figure 7C,D). 

**Figure 8:**
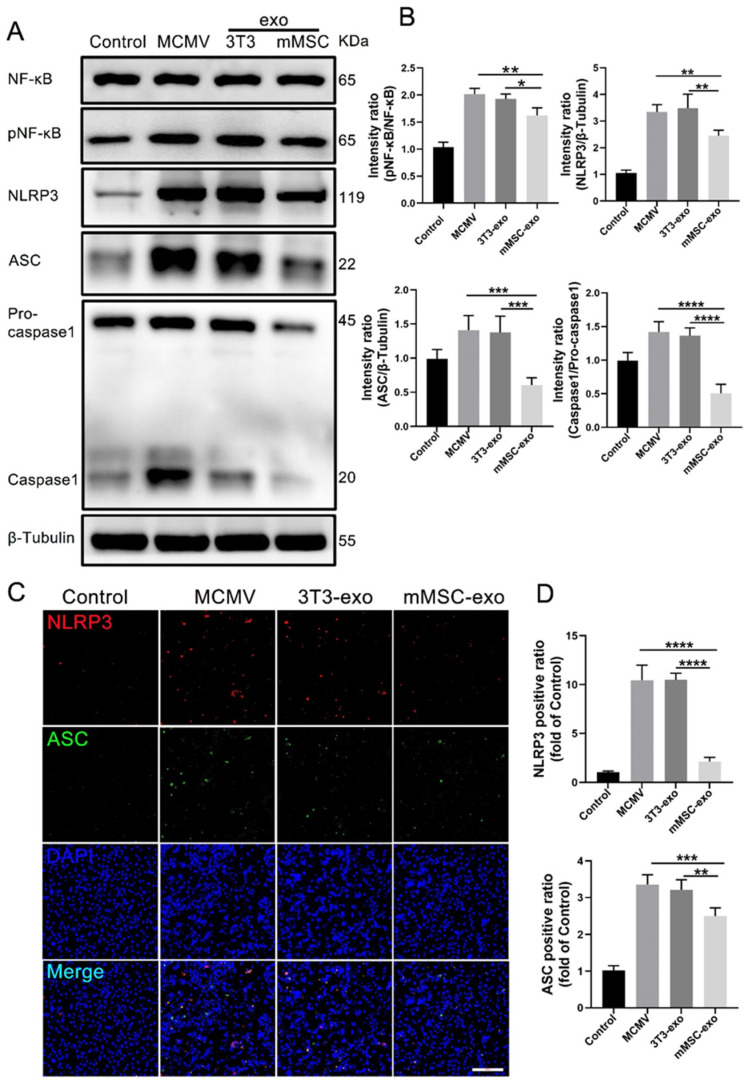
mMSC-exos inhibit NF-κB and NLRP3 signaling pathways in MCMV-infected BMDMs. (**A**) Immunoblot analysis of NF-κB, pNF-κB, NLRP3, ASC, pro-caspase-1, and caspase-1. (**B**) Proteins levels were calculated based on the signal intensities of the protein bands. (**C**) Representative images for NLRP3/ASC/DAPI immunofluorescence staining in the BMDMs were shown (NLRP3 = red, ASC = green, DAPI = blue); Scale bars: 200 μm. (**D**) Quantitative analysis of NLRP3 and ASC positive areas in the BMDMs. The integrated optical density values obtained from image J software were used to assess the expression of NLRP3 and ASC in the macrophages. Multiple group comparisons: one-way ANOVA with Tukey’s post hoc test. Data are reported as mean ± SEM, *n* = 4. * *p* < 0.05, ** *p* < 0.01, *** *p* < 0.001, **** *p* < 0.0001 compared to MCMV group.

To study the effects of mMSC-exos on the NF-κB and NLRP3 signaling pathways in vitro, we utilized BMDMs. Western blot analysis demonstrated that treatment with mMSC-exos also reduced NF-κB phosphorylation, caspase-1 activation, and the expressions of NLRP3 and ASC (Figure 8A,B). Immunofluorescence staining further supported these findings, showing decreased expression levels of NLRP3 and ASC upon mMSC-exo treatment (Figure 8C,D). These results indicate that post-infection treatment with mMSC-exos significantly attenuates the activation of NLRP3 and NF-κB in lung tissue.

## 4. Discussion

Inflammatory pathways are dysregulated in CMV pneumonia, leading to hyperinflammation characterized by macrophage activation and neutrophil recruitment [30,31,32]. The NF-κB/NLRP3 inflammasome signaling pathways play a critical role in the lung immune response, contributing to both the initiation and progression of pneumonia [7,8,33,34]. CMV can directly activate the NF-κB pathway, resulting in the expression and assembly of NLRP3, which leads to an over-release of pro-inflammatory cytokines and chemokines [7,8,33]. Therefore, these pathways are strongly correlated and form a complex network of inflammatory cascades that contribute to tissue damage [1]. Consistent with this, inhibition of the NF-κB/NLRP3 pathway has been shown to have significant therapeutic effects in murine models of CMV-pneumonitis [33]. 

As mentioned earlier, treatment with MSCs has the potential to reduce cytokine production associated with COVID-19-induced acute respiratory distress syndrome by targeting the molecular cascade of the NF-κB/NLRP3 pathway [35,36]. Like MSCs, MSC-exos have also been reported to have potent immunomodulatory effects [37,38]. The use of exosomes derived from MSCs harvested from the bone marrow has been linked to a decrease in systemic inflammatory markers, leading to a significant reduction in the cytokine storm in viral pneumonitis, including COVID-19 [13,39]. In addition to reducing systemic inflammation, the anti-inflammatory and tissue regenerative properties of MSC-exos are believed to contribute to their ability to reverse lung damage caused by viral pneumonia [40,41]. Our results suggest that MSC-exos are capable of modulating the transition of pulmonary macrophage phenotypes, as indicated by the expression of markers associated with pro-inflammatory “M1-like” states shifting towards anti-inflammatory “M2-like” states. Consistent with our findings, Willis et al. demonstrated that MSC-exos can induce macrophage polarization towards the M2 state and restore lung function in experimental bronchopulmonary dysplasia [42]. Macrophage polarization is tightly regulated by the NF-κB signaling pathway. Our in vitro results showed that MSC-exo treatment could inhibit NF-κB activation, resulting in the reduced release of pro-inflammatory cytokines and NLRP3 expression. Treatment with MSC-exos reduced the expression of cleaved caspase-1, indicating that NLRP3 inflammasome activation is also suppressed by MSC-exos. Consistent with our findings, several studies have demonstrated that MSCs and their exosomes can suppress NLRP3 inflammasome-driven inflammation [43,44]. Therefore, the anti-inflammatory effects of MSC-exos may be attributed to their inhibition of NF-κB and NLRP3 inflammasome signaling pathways.

MSCs could enhance the secretion of soluble factors, including IL-10 and transforming growth factor-beta (TGF-β), which can downregulate NF-κB activation in host cells infected with a viral agent. This, in turn, limits the overall synthesis of pro-inflammatory cytokines [45,46]. Additionally, the administration of MSCs has been shown to effectively modulate NLRP3 inflammasome activation. MSCs can secrete prostaglandin E2 (PGE2) and TGF-β, which can interrupt the NLRP3 assembly, thereby mitigating the secretion of pro-inflammatory cytokines [47]. As a result of NLRP3 inflammasome inhibition, M1 macrophages could switch to immunosuppressive (M2) phenotypes and mainly produce anti-inflammatory IL-10 instead of inflammatory IL-1β and IL-18. However, the exact mechanism of MSC-exos on their inhibition of the NF-κB/NLRP3 pathway has not been clarified, and further studies should be conducted to investigate this mechanism. 

MSC-exos have been shown to directly inhibit the replication of influenza virus and hepatitis C virus through an unknown mechanism [48,49]. However, in our study, we did not observe any inhibitory effect of MSC-exos on the viral loads in the supernatant of MCMV-infected BMDMs. We speculate that the discrepancy in results between other studies and our findings may be attributed to the different viruses being studied. Anti-infiltrating neutrophils might hinder MCMV replication through TNF-related apoptosis-inducing ligand expression [50]. The reduction in neutrophil activity resulting from MSC-exo treatment in our study might counteract the benefits of virus control. The therapeutic efficacy of mMSC-exos in CMV-pneumonitis treatment may stem entirely from their anti-inflammatory characteristics.

In summary, the therapeutic use of mMSC-exos can reduce lung inflammation and the phenotype of BMDMs associated with MCMV-induced pneumonia by specifically inhibiting NF-κB/NLRP3 inflammasome signaling. Through various mechanisms involving inhibitory mediators, we discovered that mMSC-exos can significantly alleviate the pro-inflammatory cascades of NF-κB/NLRP3. As our understanding of these phenomena continues to grow, it may lead to the development of targeted therapeutic interventions that directly target the underlying mechanisms of CMV-induced lung inflammation. This can effectively enhance outcomes for immunocompromised patients with CMV pneumonia.

## Data Availability

Data are contained within the article.
